# Antibiotics and Host-Tailored Probiotics Similarly Modulate Effects on the Developing Avian Microbiome, Mycobiome, and Host Gene Expression

**DOI:** 10.1128/mBio.02171-19

**Published:** 2019-10-15

**Authors:** Tonya L. Ward, Bonnie P. Weber, Kristelle M. Mendoza, Jessica L. Danzeisen, Katharine Llop, Kevin Lang, Jonathan B. Clayton, Elicia Grace, Jeanine Brannon, Igor Radovic, Mai Beauclaire, Timothy J. Heisel, Dan Knights, Carol Cardona, Mike Kogut, Casey Johnson, Sally L. Noll, Ryan Arsenault, Kent M. Reed, Timothy J. Johnson

**Affiliations:** a Department of Veterinary and Biomedical Sciences, University of Minnesota, Saint Paul, Minnesota, USA; b Department of Computer Science and Engineering, University of Minnesota, Minneapolis, Minnesota, USA; c Department of Animal Science, University of Minnesota, Saint Paul, Minnesota, USA; d Department of Pediatrics, University of Minnesota, Minneapolis, Minnesota, USA; e Agricultural Research Service, United States Department of Agriculture, College Station, Texas, USA; f Department of Animal and Food Sciences, University of Delaware, Newark, Delaware, USA; g Mid-Central Research and Outreach Center, University of Minnesota, Willmar, Minnesota, USA; Iowa State University

**Keywords:** antibiotic, bacteria, fungi, host, microbiota, poultry, probiotics

## Abstract

The microbiome is important to all animals, including poultry, playing a critical role in health and performance. Low-dose antibiotics have historically been used to modulate food production animals and their microbiome. Identifying alternatives to antibiotics conferring similar modulatory properties has been elusive. The purpose of this study was to determine if a host-tailored probiotic could recapitulate effects of a low-dose antibiotic on host response and the developing microbiome. Over 13 days of life, turkey poults were supplemented continuously with a low-dose antibiotic or oral supplementation of a prebiotic with or without two different probiotics (8 cage units, *n* = 80 per group). Gastrointestinal bacterial and fungal communities of poults were characterized by 16S rRNA gene and ITS2 amplicon sequencing. Localized and systemic host gene expression was assessed using transcriptome sequencing (RNA-Seq), kinase activity was assessed by avian-specific kinome peptide arrays, and performance parameters were assessed. We found that development of the early-life microbiome of turkey poults was tightly ordered in a tissue- and time-specific manner. Low-dose antibiotic and turkey-tailored probiotic supplementation, but not nontailored probiotic supplementation, elicited similar shifts in overall microbiome composition during development compared to controls. Treatment-induced bacterial changes were accompanied by parallel shifts in the fungal community and host gene expression and enhanced performance metrics. These results were validated in pen trials that identified further additive effects of the turkey-tailored probiotic combined with different prebiotics. Alternative approaches to low-dose antibiotic use in poultry are feasible and can be optimized utilizing the indigenous poultry microbiome. Similar approaches may also be beneficial for humans.

## Abstract (precis)

Alternative approaches are greatly needed to reduce the need for antibiotic use in food animal production. This study utilized a pipeline for the development of a host-tailored probiotic to enhance performance in commercial turkeys and modulate their microbiota, similar to the effects of low-dose antibiotic administration. We determined that a host-tailored probiotic, developed in the context of the commercial turkey gut microbiome, was more effective at modulating these parameters than a nontailored probiotic cocktail. Furthermore, the host-tailored probiotic mimicked many of the effects of a low-dose antibiotic growth promoter. Surprisingly, the effects of the antibiotic growth promoter and host-tailored probiotic were observed across kingdoms, illustrating the coordinated interkingdom effects of these approaches. This work suggests that tailored approaches to probiotic development hold promise for modulating the avian host and its microbiota.

## INTRODUCTION

Commercial turkey production in the United States is valued at more than $6 billion (1). Antibiotics are one of the reasons that food production animals, such as turkeys, can be grown sustainably using modern facilities. These practices are a major contributing factor to the relatively low cost of poultry meat across most of the developed world (2). The cessation of most antibiotics for growth promotion in both chickens and turkeys through the implementation of the U.S. Veterinary Feed Directive in January 2017 (3) and increasing consumer demand for antibiotic-free products are driving the need for alternative approaches to enhance performance and to prevent or reduce disease in these settings.

Prior to being banned for growth promotion purposes, many low-dose antibiotics were used in poultry production, including, but not limited to, bacitracin methylene disalicylate (BMD), chlortetracycline, virginiamycin, and flavomycin. Some of these, including BMD, continue to be used at similar concentrations for disease prevention purposes and are able to modify the microbial community of birds and prevent bacterium-induced enteritis (4–6). The mechanisms by which low-dose feed antibiotics promote overall health and performance are complex and debatable, with evidence supporting altered microbial communities resulting in increased nutrient availability, anti-inflammatory effects, and pathogen inhibition that subsequently results in enhanced performance (2).

One strategy to achieve enhanced performance without the use of antibiotics is to alter the gut bacterial community of poults early in life by mimicking the bacterial profile induced by antibiotics. Although many directly fed microbials (referred to throughout as probiotics) are commercially available, most are designed nonspecifically for use across multiple animal hosts (7). Because host-associated microbial communities can be highly specific, one could contend that probiotics designed for, or derived from, a specific host will have better efficacy. Previous work identified clear and predictable temporal succession of the turkey gut microbiome and identified several dominant bacterial taxa positively associated with flock-level performance (4). These included Lactobacillus johnsonii, Lactobacillus aviarius, group XI *Clostridium*, and the segmented filamentous bacterium “*Candidatus* Savagella” (8). Other studies have confirmed these bacterial taxa as positively correlated with poultry performance, making them strong candidates for a turkey-tailored probiotic (9, 10).

Here, a turkey-tailored probiotic blend containing dominant lactobacilli of well-performing turkeys was developed. This probiotic modulated the microbiota, mycobiota, and host gene expression of developing turkeys in a similar fashion as the low-dose feed additive BMD. The turkey-tailored probiotic had greater effect than nontailored probiotic and prebiotic administration on overall bird performance.

## RESULTS

### Identification and validation of host-specific, microbiome-tailored probiotic candidate species.

To identify representative turkey-specific strains for a probiotic blend, historically high-performing commercial and research turkey flocks were selected for isolation of bacterial species positively correlating with increased performance. The targeted bacterial genus was *Lactobacillus* due to previous associations of *L. aviarius* and L. johnsonii with the turkey microbiota and high poult performance (4, 5). From these birds, a total of 1,267 *Lactobacillus* isolates were isolated, and subsequent 16S rRNA gene sequencing identified 105 as *L. aviarius* and 116 as L. johnsonii, the targeted bacterial species (see Data Set S1 in the supplemental material). The genomes of these isolates were then sequenced and compared using core genome single nucleotide polymorphisms (SNPs) (Fig. S1).

10.1128/mBio.02171-19.1 FIG S1 Strains of Lactobacillus johnsonii (A) and *L. aviarius* (B) isolated from high-performing turkeys. Trees are based on core genome SNP differences for 116 L. johnsonii (A) and 105 *L. aviarius* (B) strains. Reference strains from chickens, humans, pigs, rats, and yogurt are shown for comparison. Orange (Tested) and dark purple (T-Pbx) strains were used in a single-strain supplementation experiment to identify strains that best colonize and enhance growth. Strains in dark purple were used in the final T-Pbx blend. (C) Colonization by potential probiotic species. Rifampin resistance was induced in each strain(s), and strains were inoculated at day of hatch into turkey poults. The *y* axis shows recovered rifampin-resistant lactobacilli at days 3, 7, and 14 postinoculation. Download FIG S1, JPG file, 0.6 MB. Copyright © 2019 Ward et al. 2019 Ward et al. This content is distributed under the terms of the Creative Commons Attribution 4.0 International license.

10.1128/mBio.02171-19.6 DATA SET S1 16S rRNA gene classification of ileal isolates collected for this study. Pairwise PERMANOVA results for differences in centroid using UniFrac distances (bacteria) or Bray-Curtis distances (fungi). Distribution of significantly (FDR, *P < *0.05) differentially expressed genes using RNA-Seq of ileum tissue, by comparison of treatment groups. Differentially expressed pathways in host ileum tissue in response to antibiotic treatment. Lists of unique and shared KEGG pathways impacted by BMD and T-Pbx treatment in the cecum and ileum. Coefficient of variation in body weights (grams), feed conversion ratios, and average daily gain (grams) in pen trial. Download Data Set S1, XLSX file, 0.2 MB. Copyright © 2019 Ward et al. 2019 Ward et al. This content is distributed under the terms of the Creative Commons Attribution 4.0 International license.

**FIG 1:**
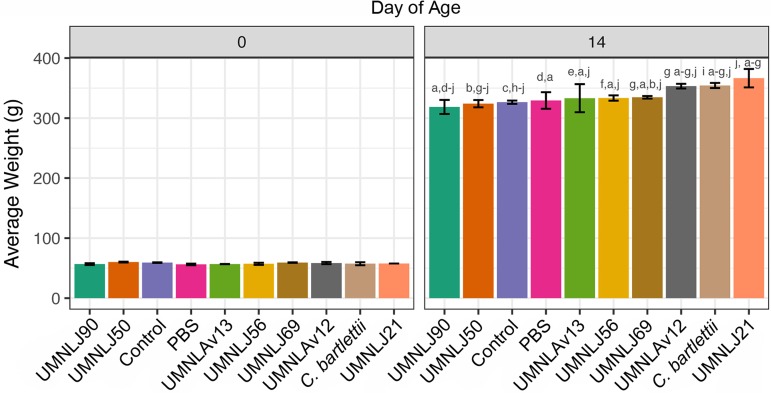
Enhanced weight gain achieved through single-strain probiotic supplementation. Each treatment represents 30 birds, with standard deviation shown. Weight differences at day 14 were tested with ANOVA and Tukey’s HSD. Bars with different letters are significantly different.

A preliminary performance/colonization experiment was performed using representative isolates from dominant phylogenetic clades, including five L. johnsonii and two *L. aviarius* strains. An ATCC strain of a group XI *Clostridium* (Clostridium bartlettii BAA-827) was also assessed because of previous correlation of this bacterial species with enhanced flock performance (4). All inoculated strains were reisolated from the poult ileum for at least 7 days following oral inoculations (Fig. S1), indicating sufficient colonization over this time period. Of the L. johnsonii strains examined, one strain (UMNLJ21) displayed increased colonization, with a 12% increase over 14 days in comparison to controls (Fig. 1, *P* = 0.04). Other strains enhanced growth with increases in body weight ranging from 2.1% to 8.5%. From this work, representatives of L. johnsonii (UMNLJ21), Lactobacillus aviarius subsp. *aviarius* (UMNLAv12), Lactobacillus aviarius subsp. *araffinosus* (UMNLAv13), and group XI *Clostridium* (human-source *C. bartlettii*) were selected as components of a turkey-tailored probiotic blend.

### The turkey microbiota develops in tight succession over time.

**FIG 2:**
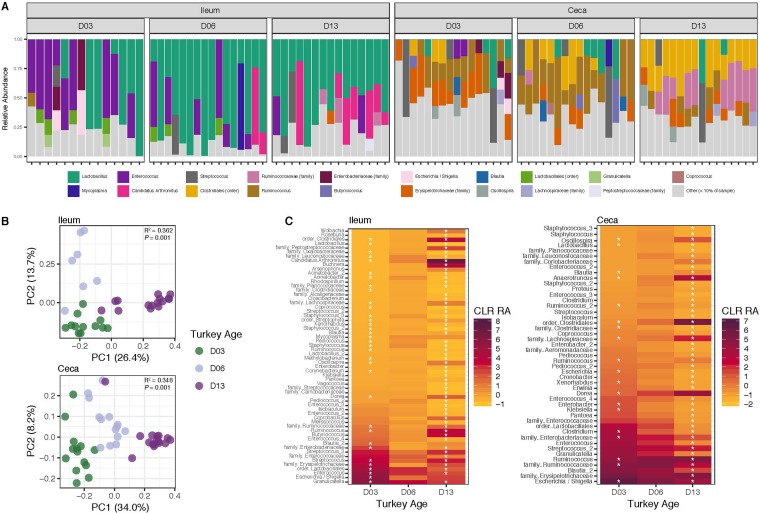
The young turkey microbiome develops over time. (A) Relative abundance of the most predominant taxa at the genus, or lowest taxonomic level denoted, per sample. “Other” represents taxa comprising less than 10% of the total relative abundance per sample. (B) Principal-coordinate analysis of unweighted UniFrac distances, colored by age of the bird. (C) OTUs significantly different in centered log-ratio-transformed relative abundance from one time point to the next. Stars denote the OTUs significantly different from that time point compared to day 6 (*P < *0.05). OTUs are labeled as their most specific taxonomic identifier available. For panels A to C, turkeys were fed only control diet. Ileum: *n* = 14, day 3; *n* = 16, day 6; *n* = 15, day 13. Cecum: *n* = 16, day 3; *n* = 16, day 6; *n* = 16, day 13.

Temporal succession of the turkey gastrointestinal microbiota has been previously demonstrated (4, 5). However, these studies focused on later-age time points, and little work has examined early microbiota succession, the focus of this study (4, 5, 11). A caged bird trial was conducted using eight replicate cages per treatment group and 10 commercial hybrid turkey poults per cage placed on day of hatch (Table 1). Birds were sampled on days 0, 3, 6, and 13 of age. For birds fed control diet, we observed within the ileum a decrease in relative abundance of *Enterococcus* and an increase in relative abundance of “*Candidatus* Savagella” over the three time points. Conversely, high proportions of *Lactobacillus* remained stable in the ileum over time (Fig. 2A). Although the ileum was generally dominated by one major taxon (*Lactobacillus*), the number of phylogenetically distinct taxa contributing to the overall microbial composition as minor members was high. In fact, alpha diversity of the ileum, measured by phylogenetic distance, was not significantly different from that of the cecum except on day 6, when the cecum had significantly lower alpha diversity (*P = *0.020; Fig. S2). In both the ileum and cecum, there was an increase in alpha diversity on day 13 compared to day 3. The ileum showed an abrupt increase from day 3 to day 6, whereas the cecum displayed the greatest increase from day 6 to day 13 (*P* = 0.0118 and *P = *6.08e−06, respectively; Fig. S2). In the cecum, *Clostridiales* and *Ruminococcaceae* increased in relative abundance by day 13 (Fig. 2A). Beta diversity measurements showed that community composition as a whole was different in both the ileum and cecum on days 3, 6, and 13, as demonstrated by the clustering of samples by day using principal-coordinate analysis (PCoA) of unweighted UniFrac distances (Fig. 2B, *P* = 0.001 and *P* = 0.001, respectively). Clustering of samples by age was due to significant differences in the relative abundances of many operational taxonomic units (OTUs) from day 3 to day 6 and from day 6 to day 13, in both tissues (Fig. 2C, *P* < 0.05). In the ileum, clustering of samples by day was less clear using weighted UniFrac or Bray-Curtis distances, demonstrating that the compositional differences between days are driven mostly by minor taxa that are phylogenetically distinct (Fig. S2). In the cecum, clustering of samples by day was clear regardless of the beta diversity metric used, demonstrating that both major and minor members of the cecal community contribute toward the overall shifts in the cecal microbiota over time (Fig. S2). These results indicate discernible bacterial community succession in the ileum and cecum during the first 2 weeks of the life of the turkey, which is the most common time frame for the use of probiotic products in poultry (10).

**TABLE 1:** Experimental design of turkey caged trial conducted over 13 days

Group	Description	Application	No. of replicates (total no. of birds)
Control	Negative control, no treatment	None	8 (80)
Prebx	Supplemented with GroGel	Daily supplement	8 (80)
FM-B11	Prebx + commercial probiotic	With Prebx	8 (80)
T-Pbx	Prebx + turkey-specific probiotic	With Prebx	8 (80)
BMD	Bacitracin methylene disalicylate, 50 g/ton	Continuous in feed	8 (80)

10.1128/mBio.02171-19.2 FIG S2 The turkey microbiome develops over time. (A) Alpha diversity (PD whole tree) for turkeys over time, *P* value reported. A Loess smoothed spline is added to visualize the abrupt increase in alpha diversity in the ileum and delayed increase in alpha diversity in the cecum. (B) Principal-coordinate analysis of unweighted UniFrac, weighted UniFrac, and Bray-Curtis distances of ileum microbiome samples, colored by age of the bird. (C) Principal-coordinate analysis of unweighted UniFrac, weighted UniFrac, and Bray-Curtis distances of cecum microbiome samples, colored by age of the bird. Download FIG S2, JPG file, 0.5 MB. Copyright © 2019 Ward et al. 2019 Ward et al. This content is distributed under the terms of the Creative Commons Attribution 4.0 International license.

### Impact of antibiotics on the microbiota.

**FIG 3:**
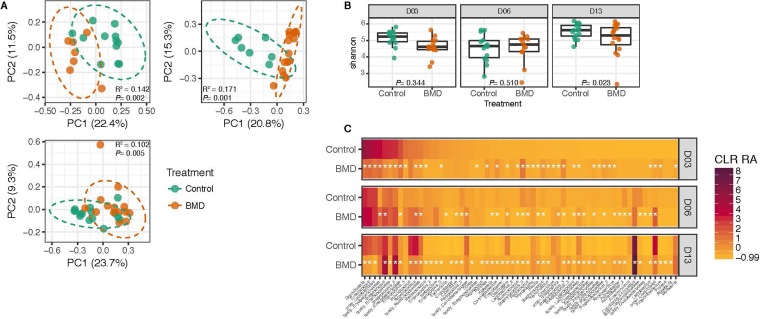
Antibiotics disrupt the turkey ileum microbiome. (A) Principal-coordinate analysis of unweighted UniFrac distances of the turkey ileum microbiome, colored by treatment (teal, control; orange, antibiotic treatment [BMD]). (B) Alpha diversity (Shannon index) for control and BMD-treated turkeys with *P* values reported. (C) OTUs significantly different in centered log-ratio-transformed relative abundance (CLR RA) in BMD versus control birds, by time point. Stars denote *P < *0.05. OTUs are labeled as their most specific taxonomic identifier available. For panels A to C, numbers were as follows: day 3, *n* = 15, BMD; *n* = 14, control; day 6, *n* = 15, BMD; *n* = 16, control; day 13, *n* = 15, BMD; *n* = 15, control.

To determine how low-dose in-feed antibiotics alter microbiome development, we compared the microbiomes of turkeys fed a standard diet (control) or a standard diet containing BMD at a continuous low-dose concentration of 50 g/ton in feed, a concentration indicated and typically used for increased weight gain and improved feed efficiency (NADA 46-592, U.S. Food and Drug Administration). In the ileum, BMD caused a change in community composition on days 3, 6, and 13, with the greatest change induced on day 6 (Fig. 3A). This change, however, was not accompanied by a change in alpha diversity, except on day 13, when BMD-treated turkeys had significantly lower alpha diversity than controls (*P = *0.023, Fig. 3B). Community composition shifts throughout development were due to significant changes in the relative abundance of numerous OTUs on each day (*P < *0.05, Fig. 3C).

Similar antibiotic-induced changes were seen in the cecum microbiome over time, including community shifts in the microbiome during each time point, as demonstrated by significant clustering of samples by treatment group using PCoA of unweighted UniFrac distances (*P < *0.002, Fig. S3). Alpha diversity was not altered in the cecum by antibiotic intake, except on day 3 of life, when BMD-treated turkeys had significantly lower alpha diversity than controls (*P = *0.013, Fig. S3). OTUs in the cecum were only significantly different in relative abundance in BMD-treated turkeys compared to controls on day 13 of life (*P < *0.05, Fig. S3).

10.1128/mBio.02171-19.3 FIG S3 Antibiotics disrupt the turkey cecum microbiome, and microbiome-induced shifts are time specific. (A) Principal-coordinate analysis of unweighted UniFrac distances of the turkey cecum microbiome, colored by treatment (control and antibiotic treatment [BMD]). (B) Alpha diversity (Shannon index) for control and BMD-treated turkeys with *P* value reported. (C) OTUs significantly different in centered log-ratio-transformed relative abundance (CLR RA) in BMD-treated versus control birds, by time point. Stars denote *P < *0.05. OTUs are labeled as their most specific taxonomic identifier available. For panels A to C, numbers were as follows: day 3, *n* = 15, BMD; *n* = 16, control; day 6, *n* = 16, BMD; *n* = 16, control; day 13, *n* = 16, BMD; *n* = 16, control. (D and E) Principal-coordinate analysis of unweighted UniFrac distances of the turkey ileum (D) and cecum (E) microbiome for each time point, colored by treatment: control, BMD, turkey probiotic (T-Pbx), commercial probiotic (FM-B11), and prebiotic (Prebx). Download FIG S3, JPG file, 1.5 MB. Copyright © 2019 Ward et al. 2019 Ward et al. This content is distributed under the terms of the Creative Commons Attribution 4.0 International license.

### Tailored probiotics mimic antibiotic-induced microbiome changes.

**FIG 4:**
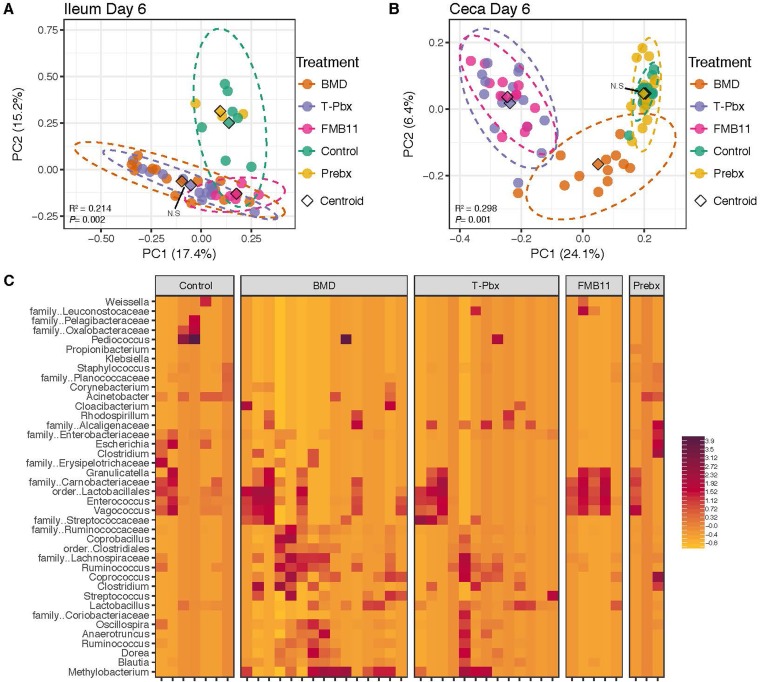
Antibiotics and probiotics similarly alter the turkey microbiome. Principal-coordinate analysis of unweighted UniFrac distances of the turkey ileum (A) and cecum (B) microbiome, colored by treatment: control, antibiotic (BMD), turkey-tailored probiotic (T-Pbx), commercial probiotic (FM-B11), and prebiotic (Prebx). Differences in centroids by treatment (denoted by a diamond) were tested by PERMANOVA, with *R*^2^ and *P* values reported. Pairwise PERMANOVA was also performed on each treatment pair, with insignificant differences in centroids (*P > *0.05) denoted by N.S. Full pairwise comparison results are listed in Data Set S1. (C) Centered log-ratio-transformed relative abundances of minor contributing OTUs in the ileum (less than 10%).

To test whether probiotics could induce changes in the microbiome similar to those induced by antibiotic administration, turkeys were supplemented with a turkey-tailored probiotic (T-Pbx) or a commercially available probiotic, FloraMax-B11 (FM-B11; Novozymes, Bagsvaerd, Denmark) (11). FM-B11 is a blend of two poultry-source lactobacillus strains not selected for host-adaptive properties or correlations with performance. These groups were compared with the prebiotic used to deliver the probiotics (Prebx), as well as with those fed the antibiotic (BMD) and control feed (control). Similar to antibiotic administration, the impact of probiotics on the ileum microbiome was greatest on day 6 (*P *= 0.001, *R*^2^ = 0.275, Fig. S3). On day 6, the BMD- and T-Pbx-fed animals had indiscernible microbiome compositions (unweighted UniFrac distances, *P = *0.107, Fig. 4A). Probiotic- and BMD-treated turkeys had microbiomes different than Prebx and control turkeys, which were indiscernible from one another (*P* < 0.01, Fig. 4A and Data Set S1). The greatest impact on the cecum microbiome was also seen on day 6 compared to earlier and later time points (*P = *0.001, *R*^2^ = 0.306, Fig. S3). In the cecum on day 6, neither T-Pbx or FM-B11 was able to recapitulate community composition changes induced by BMD administration (Fig. 4B). The probiotics did, however, alter the cecum microbiome distinctly to differentiate T-Pbx and FM-B11 turkeys from Prebx and controls (Fig. 4B and Data Set S1). Cecum microbiomes of control and Prebx-fed turkeys on day 6 were not significantly different (unweighted UniFrac distances, *P = *0.113, Fig. 4B). Microbiome changes induced on day 6 in the ileum involved minor members of the bacterial community, as demonstrated by plotting OTUs consisting of less than 10% of the total relative abundance of a sample (Fig. 3C and Fig. 4C).

### Tailored probiotics mimic antibiotic-induced mycobiome changes in the ileum.

**FIG 5:**
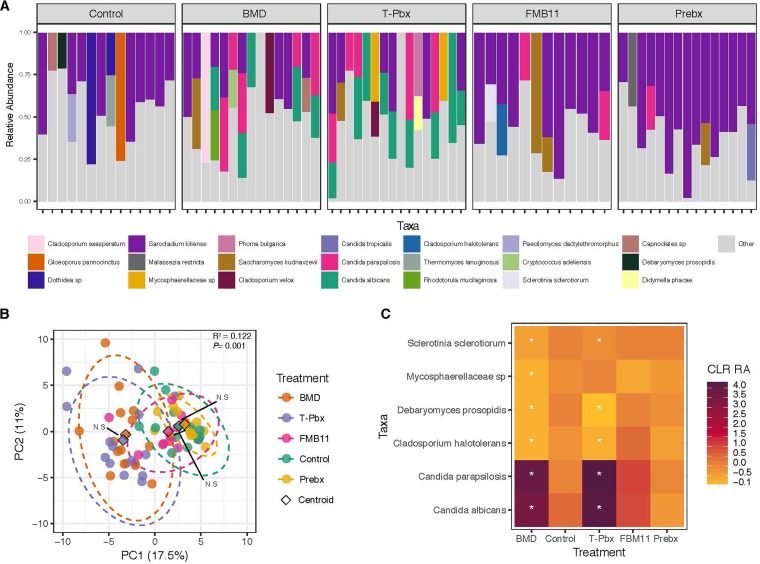
Antibiotics and probiotics similarly alter the turkey mycobiome on day 6. (A) The relative abundance of the most predominant fungal taxa at the species, or lowest taxonomy level denoted, per sample. “Other” represents taxa comprising less than 20% of the total relative abundance per sample. (B) Principal-coordinate analysis of Bray-Curtis distances, colored by treatment. Differences in centroids by treatment (denoted by a diamond) were tested by PERMANOVA, with *R*^2^ and *P* values reported. Pairwise PERMANOVA was also performed on each treatment pair, with insignificant differences in centroids (*P > *0.05) denoted by N.S. Full pairwise comparison results are listed in Data Set S1. (C) OTUs significantly different in centered log-ratio-transformed relative abundance (CLR RA) from one time point to the next. Stars denote OTUs significantly different in that treatment compared to its control treatment (*P < *0.05). OTUs are labeled as their most specific taxonomic identifier available. For panels A to C, numbers were as follows: *n* = 14, control; *n* = 15, BMD; *n* = 16, T-Pbx; *n* = 12, FM-B11; *n* = 15, Prebx.

To test whether probiotics and antibiotics induced similar changes in the mycobiome (fungal community), the ileum mycobiome was characterized using ITS2 amplicon sequencing. Fungal alpha diversity increased over time from day 3 to day 13 (*P = *0.007, Fig. S4). The most predominant fungal taxon in the turkey ileum, regardless of treatment, was Sarocladium kiliense (Fig. 5A), and alpha diversity was not significantly modified by any treatment except T-Pbx versus Prebx control on days 6 and 13 (Fig. S4, *P = *0.020 and *P = *0.010, respectively). Treatment impacts on the overall mycobiome were most distinct on day 6 (Fig. S4), when the BMD and T-Pbx turkeys were indistinguishable (Bray-Curtis distances, *P = *0.594, Fig. 5B), controls and FM-B11 were indistinguishable (*P = *0.301, *P = *0.297, and *P = *0.234, respectively; Fig. 5B), and yet BMD and T-Pbx were significantly different from the controls and FM-B11 (*P = *0.001). Compared to respective controls, both BMD and T-Pbx treatment caused significant increase in the relative abundance of OTUs indicated as Candida parapsilosis and Candida albicans and a significant decrease in the relative abundance of OTUs indicated as Sclerotinia sclerotiorum, Debaryomyces prosopidis, and Cladosporium halotolerans in the ileum on day 6 (*P < *0.05, Fig. 5C). These differences were not seen at the earlier or later time points (Fig. S4). Also on day 6, treatment-induced changes in the ileum mycobiome were significantly correlated with changes in the microbiome, as tested by Procrustes analysis of principal coordinates of bacterial unweighted UniFrac distances and fungal Bray-Curtis distances (Fig. S5, *P = *0.033, *M*^2^ = 0.875).

10.1128/mBio.02171-19.4 FIG S4 Mycobiome differences and fungal taxa differences are time specific. (A) Fungal alpha diversity (Shannon index) in the ileum of control turkeys over time, *P* value reported if significant. A Loess smoothed spline is added to visualize the overall increase in alpha diversity over time. (B) Fungal alpha diversity (Shannon index) in the ileum of turkeys given feed supplemented with antibiotics (BMD), a turkey probiotic (T-Pbx), a commercial probiotic (FM-B11), a prebiotic (Prebx), or nothing (control) with *P* value reported if significant. (C) Principal-coordinate analysis of Bray-Curtis distances of the turkey ileum mycobiome, colored by treatment: control, BMD, turkey probiotic (T-Pbx), commercial probiotic (FMB11), and prebiotic (Prebx). Differences in centroids by treatment were tested by PERMANOVA, with *R*^2^ and *P* value reported. (D) A heat map of the centered log-ratio-transformed relative abundance (CLR RA) of fungal OTUs. OTUs are labeled as their most specific taxonomic identifier available. Download FIG S4, JPG file, 1.6 MB. Copyright © 2019 Ward et al. 2019 Ward et al. This content is distributed under the terms of the Creative Commons Attribution 4.0 International license.

10.1128/mBio.02171-19.5 FIG S5 Fungi are correlated with bacteria in the ileum, and antibiotics influence the turkey transcriptome. (A) Procrustes analysis of principal coordinates from bacterial unweighted UniFrac distances and fungal Bray-Curtis distances from day 6 in the ileum. Samples are colored by treatment: control, antibiotic (BMD), turkey-tailored probiotic (T-Pbx), commercial probiotic (FM-B11), and prebiotic (Prebx). *M*^2^ and *P* value from *n* = 999 permutations are reported. (B and C) Bacterial OTUs significantly correlating with fungal OTUs within antibiotic (B)- or turkey-tailored probiotic (C)-treated animals on day 6 in the ileum. Correlations shown are *P* < 0.25 following false-discovery rate correction. Significant correlation *P* values are reported (Pearson’s correlation). OTUs are labeled as their most specific taxonomic identifier available. (B) *n* = 14, BMD. (C) *n* = 13, T-Pbx. (D) Principal-coordinate analysis of Euclidean pathway expression distances, colored by treatment. Differences in centroids by treatment (denoted by diamonds) were tested by PERMANOVA, with *R*^2^ and *P* value reported. Pairwise PERMANOVA was also performed on each treatment pair, with insignificant differences in centroids (*P > *0.05) denoted by N.S. (E) Bacterial OTUs significantly correlating with reactome pathways on day 6 in the ileum. The star denotes *P* < 0.05 following false-discovery rate correction (Pearson’s correlation; samples from all treatments were used). OTUs are labeled as their most specific taxonomic identifier available. For both panels, numbers were as follows: *n* = 3, control; *n* = 4, BMD; *n* = 3, T-Pbx; *n* = 2, FM-B11; *n* = 1, Prebx. Download FIG S5, JPG file, 0.7 MB. Copyright © 2019 Ward et al. 2019 Ward et al. This content is distributed under the terms of the Creative Commons Attribution 4.0 International license.

### Host phenotypes are correlated with microbiome changes and treatment.

**FIG 6:**
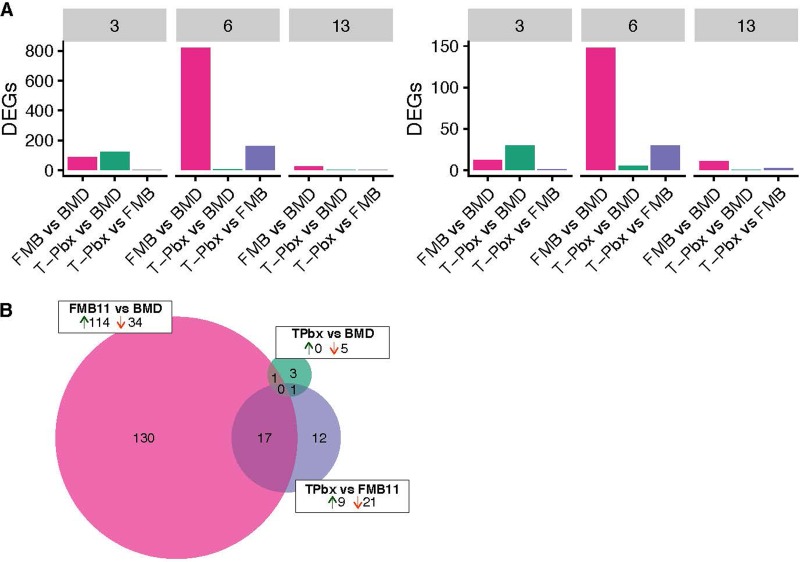
Antibiotics and probiotics can modulate ileal gene expression on day 6 of life. (A) Distribution of significant differentially expressed genes (*P < *0.05) in the turkey at |log_2_FC| > 1.0 (left) and |log_2_FC| > 2.0 (right) according to pairwise tests with false-discovery rate correction, faceted by day of life. (B) Shared and unique differentially expressed genes in the turkey ileum on day 6 at |log_2_FC| > 2.0. Circle size is proportional to the number of genes, and direction of expression change (↑ or ↓) is given.

To determine if antibiotic- and probiotic-induced shifts in the microbiome led to an altered host phenotype, transcriptome sequencing (RNA-Seq) was used to measure host transcript levels in the ileum. Pairwise treatment comparisons resulted in 2 to 821 significantly (false-discovery rate [FDR] *P* value < 0.05) differentially expressed genes (DEGs) at |log_2_FC| ≥ 1.0, where FC is fold change, and 0 to 148 DEGs at |log_2_FC| ≥ 2.0 (Fig. 6A and Data Set S1). On day 3 of life, the most dissimilar transcriptomic profiles were T-Pbx turkeys compared to BMD turkeys with 121 (|log_2_FC| ≥ 1.0) and 30 (|log_2_FC| ≥ 2.0) DEGs, although FM-B11 also had a dissimilar profile compared to BMD (86 and 12 (|log_2_FC| ≥ 1.0 and |log_2_FC| ≥ 2.0, respectively). However, on day 6, T-Pbx versus BMD turkeys had converging transcriptomic profiles (7/5 DEGs), and T-Pbx versus BMD became even more similar on day 13 (2/0 DEGs). In contrast, BMD turkeys were distinct from FM-B11 turkeys on day 6 (821/148 DEGs) and became more similar to BMD turkeys on day 13 (24/11, Data Set S1). Of the five DEGs found between T-Pbx and BMD turkeys, only one was also found in the FM-B11-versus-BMD comparison, further highlighting the distinctions in antibiotic-like shifts for the turkey-tailored probiotic compared with larger numbers of unique differences for the FM-B11 treatment (Fig. 6B). In greater support of this, of the 30 DEGs found between FM-B11 and T-Pbx turkeys, 17 (57%) were shared with FM-B11 versus BMD turkeys (Fig. 6B).

The greatest fold enrichments for BMD treatment on day 6, and also for T-Pbx treatment, were observed for gene ontology (GO) biological processes indicative of angiogenesis, mTOR signaling, and morphogenesis/development (Data Set S1). The greatest upregulation in BMD-treated birds was observed for fibroblast growth factor 14 (*FGF14*) and laminin subunit alpha-2-like (*LOC109366368*), genes with broad mitogenic and cell survival properties implicated in development, cell growth, and morphogenesis (12). The greatest downregulation occurred in loci with diverse functions (ankyrin repeat domain 24 [*ANKRD24*] locus, potassium calcium-activated channel subfamily M regulatory beta subunit 4 [*KCNMB4*], activin A receptor type 1C [*ACVR1C*], C-type lectin domain family 2 member B [*BLEC1*, *LOC100538559*], and several uncharacterized ncRNA loci [*LOC104913259*, *LOC104916736*, and *LOC109368595*]), including control of smooth muscle tone (13); signaling molecules with high expression in adipose and digestive system tissues (14), correlating with measures of body fat, carbohydrate metabolism, and lipids (15); and cell adhesion, immune response to pathogens, and apoptosis (16). Significant enrichment was also seen for genes involved in protein-lysine 6-oxidase activity (GO:0004720, LOX), acting in posttranslational oxidative deamination of peptidyl lysine residues (17). Similar shifts were observed for T-Pbx treatment.

**FIG 7:**
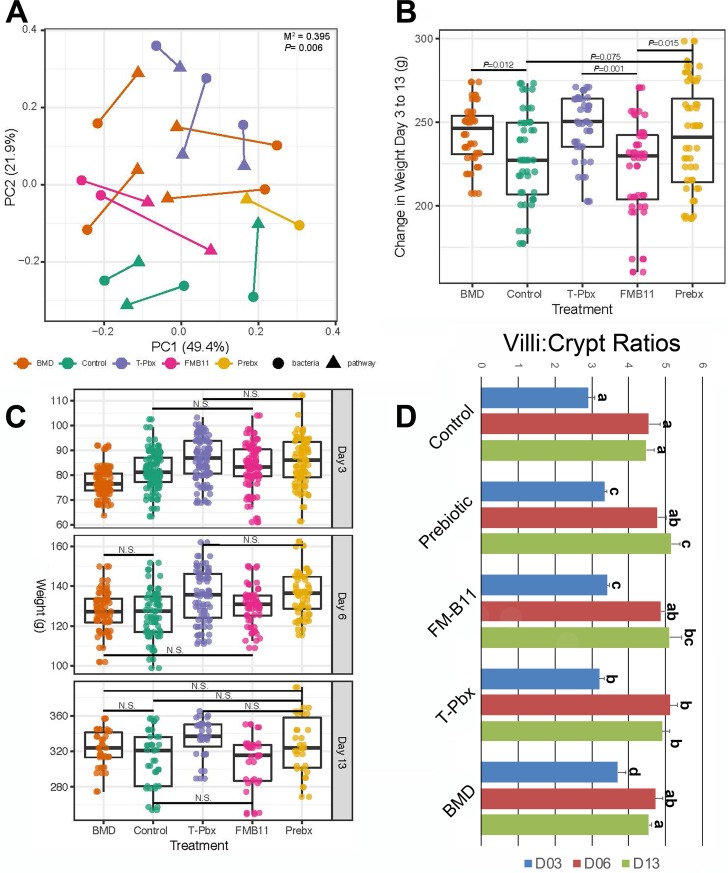
Host phenotypes are correlated with microbiome shifts and treatment. (A) Procrustes analysis of principal coordinates from bacterial unweighted UniFrac distances and reactome pathway Euclidean distances. Samples are colored by treatment: control, antibiotic (BMD), turkey-tailored probiotic (T-Pbx), commercial probiotic (FM-B11), and prebiotic (Prebx). *M*^2^ and *P* values from *n* = 999 permutations are reported. (B) Change in total body weights from day 3 to day 13 for turkeys separated by treatment and tested via Student’s *t* test with *P* values reported. Numbers per treatment: control, *n* = 37; BMD, *n* = 38; T-Pbx, *n* = 36; FM-B11, *n* = 36; Prebx, *n* = 34. (C) Weights for each turkey on days 3, 6, and 13 of life, separated by treatment. Differences in weight by treatment were tested for by using a Student *t* test. N.S. represents *P* values of <0.05. (D) Villus height/crypt depth ratios averaged by treatment group in the ileum across treatment groups. Letters denote statistical significance (*P < *0.05).

There were overall shifts in reactome pathways using the transcriptome of BMD and T-Pbx turkeys compared to controls (*P < *0.05, Fig. 7A), yet FM-B11 did not significantly differ from controls. Overall, pathways were also significantly correlated with shifts in the bacterial microbiome by Procrustes analysis of principal coordinates of bacterial unweighted UniFrac distances and reactome Euclidean distances (Fig. 7A, *P* = 0.006, *M*^2^ = 0.395), confirmed by a Mantel test using the full distance matrices of the reactome and unweighted UniFrac distances (*P = *0.002, *R* = 0.340).

**FIG 8:**
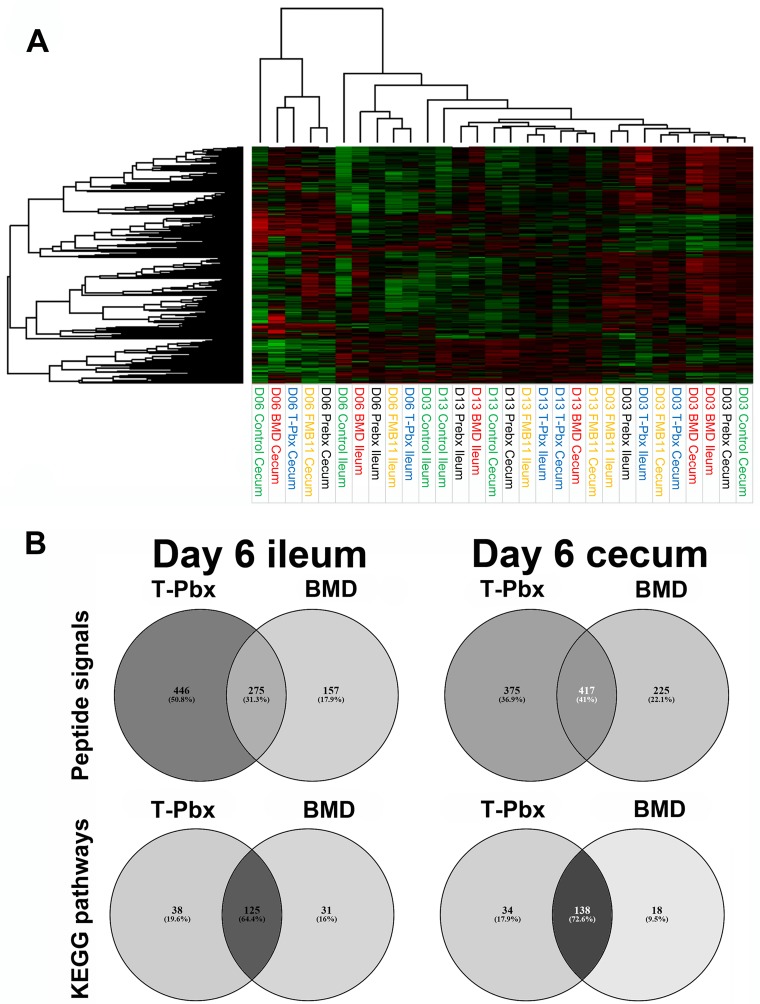
(A) Peptide phosphorylation kinome profiles display clustering patterns by treatment, and turkey weight is loosely associated with treatment. Phosphorylation data for each peptide target site are presented, relative to control, on a heat map; red is increased phosphorylation, and green is decreased phosphorylation. Each condition is shown on the *x* axis. The connecting lines above the heat map show the relative similarity clustering of the different conditions described by the length of the lines. (B) Unique and shared effects of T-Pbx and BMD at the peptide signal (top) and KEGG pathway (bottom) levels, compared to their respective controls.

Avian-specific kinome peptide arrays were then used to generate kinome profiles (kinotypes) for ileum and cecum tissues across treatments from days 3, 6, and 13 (Fig. 8), representing active kinases in the tissue sample. Heat map and cluster analysis, in agreement with the microbiome data, showed that kinotypes clustered primarily by bird age. Within age clusters, similarities were predominantly by tissue type. Again, day 6 showed the greatest separation in response, with clear clustering of day 6 samples. Within day 6 cecum clusters, pairs of T-Pbx/BMD and FM-B11/Prebx showed the greatest similarity, agreeing with microbiome data showing that T-Pbx yielded effects similar to the antibiotic treatment. In the day 6 and 13 ileum samples, and day 3 cecum samples, the two probiotics clustered more closely.

Kinome data were analyzed at levels of individual signals and KEGG pathway effects (Fig. 8B). Because of similarities in effects between T-Pbx and BMD treatments, shared and unique effects of these treatment groups were assessed relative to respective control groups. Of the significant effects, 31.3% and 41% of T-Pbx and BMD treatment peptide signals were similarly impacted in the ileum and cecum, respectively. At the KEGG pathway level, 64.4% and 72.6% of the pathways were similarly impacted in the ileum and cecum, respectively, indicating that overall functional effects of BMD and T-Pbx shared substantial overlap that may be conferred through different individual peptide effects. In the ileum and cecum, 125 and 138 KEGG pathways were significantly impacted in both BMD and T-Pbx treatments, respectively (Data Set S1). Of these pathways, 123 overlapped between ileum and cecum tissue. Notably, the most substantially impacted pathways (based upon FDR *P* value and number of genes within the pathway impacted) included those involved in nutrient utilization, metabolism, cell growth and differentiation, cancer pathways, cell layer junctions, immune response, and response to pathogens. Fewer pathways were identified as unique to either BMD or T-Pbx treatment (Fig. 8B). Of note, in the T-Pbx treatment group, a unique effect compared to the BMD treatment group was the enhancement of pathways involved in interleukin 17 (IL-17) signaling, Th17 cell differentiation, and Th1 and Th2 cell differentiation. This was in contrast to shared effects of BMD and T-Pbx treatments on pathways involved in natural killer cell-mediated cytotoxicity, T and B cell receptor signaling, tumor necrosis factor (TNF) signaling, Fc epsilon R1 and Fc gamma R signaling, and leukocyte migration (Data Set S1).

BMD, T-Pbx, and Prebx caused a significant increase in the amount of weight gained by turkeys from day 3 to day 13 compared to the controls and FM-B11 turkeys in the caged bird trials (Fig. 7B, *P* < 0.05). The BMD, T-Pbx, and Prebx turkeys, however, had no significant difference in weight gain from one another (Fig. 7B, *P* > 0.05). Only T-Pbx-treated turkeys trended higher at days 3 and 6 and were significantly heavier at day 13 (Fig. 7C, *P* = 0.001 and *P* *= *6.63e−5, respectively), compared to controls and FM-B11. As a phenotypic measure of gut development, villus height/crypt depth ratios were significantly higher for T-Pbx-treated turkeys than controls across all time points (Fig. 7D).

### Effects of host-specific probiotics on performance and the microbiota are reproducible in pen studies.

To validate if effects observed in short-term caged bird experiments were translatable and reproducible, a 15-week pen trial was performed. In this study, 7 pen replicates per treatment were included with 24 turkeys per pen. In addition to testing the effects of T-Pbx compared with negative controls, T-Pbx was combined with in-feed application of 1% lactose or a commercial yeast cell wall product in two additional treatment groups because of previous work suggesting synergistic impacts of combining specific combinations of prebiotic and probiotic (18). Significant enhancements in body weights were observed for all three treatment groups (T-Pbx, T-Pbx plus 1% lactose, and T-Pbx plus yeast cell wall product) compared to the negative control, at three sampling time points, and body weights and average daily gain trended higher in these treatments throughout the study (Table 2 and Data Set S1). Also, bird-to-bird variation was significantly reduced in treatment groups at days 21 and 28 of age. Furthermore, feed conversion ratio (FCR) was significantly enhanced for treatment groups from 0 to 2 weeks of age. Collectively, these results indicate a reproducible performance-enhancing effect for T-Pbx which was amplified by the addition of a yeast-based or sugar prebiotic.

**TABLE 2:** Average total body weights of turkeys in pen trials conducted over 15 weeks

Treatment	Avg total body wt (g) at day:							
	0	7	14	21	42	63	84	105
Control	64.2	171.5	343.0	692.9	2,419.1	5,370.1	9,129.3	13,545.0
TJ-Pbx	64.8	168.7	347.6	709.2	2,439.5	5,455.0	9,500.0	13,685.0
TJ-Pbx plus 1% lactose	65.0	177.0	359.3	729.6	2,473.1	5,509.8	9,501.4	13,848.0
TJ-Pbx plus yeast	65.0	174.0	361.7	728.0	2,468.9	5,469.6	9,425.0	13,677.0

*P* value	0.54	0.53	<0.001	<0.001	0.33	0.42	<0.001	0.12

## DISCUSSION

This study provides insights into the interactions occurring between the bacterial microbiome, mycobiome, and localized host gene expression using the turkey as a model. Using low-dose antibiotic administration via feed, we found that a targeted approach to probiotic design can effectively mimic some of the changes induced by low-dose antibiotics. These effects were not induced with a nontargeted probiotic approach, highlighting the importance of considering host-tailored strains in the context of the host’s microbiome when developing probiotics with the goal of modulating microbiota and the host. We found that, even very early in the turkey poult’s life, bacterial succession is strictly age dependent, consistent, and reproducible (Fig. 2). This holds true for both the ileum and the cecum.

Others have reported high variability in the poultry microbiome (19, 20), possibly due to confounding factors such as environment, season, genetic line, bird type, and diet. However, these variations are overridden by the strong and consistent effect of age, validated through the present study which controlled for these factors. We found that “*Candidatus* Savagella” appeared earlier and in higher abundance in BMD and T-Pbx treatment groups, similar to what has been previously found in turkeys (4), and our finding highlights the possible importance of segmented filamentous bacteria such as “*Candidatus* Savagella” at priming the immune system and promoting diversification of the healthy microbiome in the gut (21). In the cecum, there is a trend from facultative anaerobes toward a diverse anaerobic bacterial composition, likely reflecting physical changes occurring in the gut during the first weeks of age. The concept of an orchestrated succession of the microbiota has been observed in other animals, including humans (22–24). Irrespective of environment, evidence would suggest that this occurs across animals in a reproducible means.

A surprising finding of this work was that parallel and correlated shifts were identified between the bacterial microbiome, mycobiome, and host response. These shifts were induced by both antibiotic administration and turkey-tailored probiotic administration. To our knowledge, this study provides novel evidence for coordinated interkingdom relationships between host, bacteria, and fungi occurring at the gut level. While we cannot delineate cause and effect in this study, it is clear that the microbial players in the gut are interacting either with one another or at least with the host, and this is reflected by parallel shifts during modulation. This knowledge reinforces the notion that use of live microbial modulations has the potential to impact not only the target kingdom (i.e., bacteria) but also other kingdoms of life within a particular host body habitat (i.e., fungi). This also underscores that changes observed in host gene expression upon modulation by a probiotic or directly fed microbial are not simply the effects of that product itself, nor the bacterial community itself, but rather the community state as a whole.

The mechanisms of the effects of growth-promoting antibiotics on production animal performance have been studied for many years (25, 26). While pathogen inhibition is long established as a primary contributor to antibiotic growth promoter efficacy, other mechanisms have been proposed, including anti-inflammatory and immunostimulatory effects at the gut level. This study supports that proposal, as numerous host pathways associated with immunostimulation and cell barrier enhancement were enriched under BMD treatment, in addition to pathways associated with cell growth/proliferation and pathogen response. Surprisingly, most of the host pathways affected by BMD treatment were similarly impacted by host-tailored probiotic treatment but not by non-host-tailored probiotic treatment. Furthermore, additional pathways were uniquely impacted by the host-tailored probiotic treatment that suggest additional mechanisms of immunostimulation at the gut level involving Th17 and IL-17. We observed that treatment with T-Pbx enhanced the relative abundance of segmented filamentous bacteria in the ileum, a phenomenon which we have previously correlated with high-performing turkey flocks (4). These bacteria have been shown to induce the accumulation of Th17 cells in the small intestines of various animals (27), which play an important role in host defense against fungal and bacterial pathogens (28). Our data suggest that T-Pbx colonizes the developing turkey poult and not only induces positive effects on the commensal resident microbiota but also in turn confers immunostimulatory effects that collectively result in benefits for the turkey in gut development and barrier protection. This underscores the possible benefits of utilizing host-adapted bacteria as probiotics, particularly if they are identified using a pipeline approach such as that employed here.

### Conclusion.

The landscape of food animal production is rapidly changing and is driven by consumer pressures and government regulations. These changes are in response to justifiable concerns about the role of animal agriculture in the realm of antimicrobial resistance. Animal agriculture worldwide is therefore at a crossroads where alternative approaches are needed if we are to sustain current production. Here, we demonstrate that custom approaches to probiotic development can mimic some of the longstanding positive effects of low-dose antibiotics in animal feed. In concept, this provides promise that feasible solutions using live microbials are capable of promoting beneficial results and sustainability in animal agriculture. These solutions, however, will be complex in that custom approaches need to be tailored to specific farms, animal types, host genetics, production goals, and local challenges. Similarly, custom approaches in humans need to carefully consider confounding factors and unexpected variables, such as diet, geography, climate, and pathogen pressures. Therefore, although the baseline data presented here are promising, more work is required before the world of customized microbial medicine is realized.

## MATERIALS AND METHODS

### Animal experiment statement.

All animal experiments were conducted at the University of Minnesota following guidelines for ethical and humane treatment of animals, under protocol 1608-34049A.

### *Lactobacillus* isolation from turkey ilea.

Bacterial isolates were collected from two high-performing turkey flocks in Minnesota and Iowa. Sampling of flocks was performed weekly until 42 days of age and every other week through 12 weeks of age. At each sampling time point, 10 birds per flock were randomly selected and humanely euthanized. Ileum homogenates were serially diluted in phosphate-buffered saline and plated onto *Lactobacillus* selection or De Man, Rogosa, and Sharpe (MRS) agar and then incubated overnight at 37°C. Isolated colonies were selected for further study.

Isolates were subjected to a *Lactobacillus*-specific PCR as previously described (29). For *Lactobacillus*-positive isolates, hypervariable regions 1 to 5 of the 16S rRNA gene were amplified using the 27F and 926R primers (30). Amplicons were Sanger sequenced, and *Lactobacillus* species were identified by BLASTN query against the NCBI reference database. From this analysis, L. johnsonii and *L. aviarius* were selected for further study. Once isolated, L. johnsonii was grown under aerobic conditions, whereas *L. aviarius* required growth under strictly anaerobic conditions (5% CO_2_, 5% H_2_, 90% N_2_) (31). DNA was extracted from each isolate using the DNeasy Blood and Tissue kit according to the manufacturer’s instructions (Qiagen, Valencia, CA).

### Isolate sequencing and phylogenetic analysis.

Illumina sequencing libraries were constructed using the Nextera DNA library prep kit, with dual-end indexing (Illumina, San Diego, CA, USA). Libraries were sequenced on Illumina HiSeq using HiSeq Rapid SBS kit V2 with 2- by 150-bp paired-end (PE) chemistry. Following sequencing, fastq files were quality trimmed using Trimmomatic (32). Groomed sequences were mapped to appropriate reference genomes using BWA aligner (33) with a minimum fraction of 0.9. Variants were called using CLC Genomics Workbench (version 10) using default parameters for haploid genomes. The resulting SNP matrix was analyzed using Maximum Parsimony methods in MEGA7 (34) with 100 bootstrap replicates. Phylogenetic trees were visualized using iTOL (35).

### Single-strain colonization experiments.

All culturing was performed using anaerobic conditions (5% CO_2_, 5% H_2_, 90% N_2_). For single-strain colonization experiments, wild-type strains were cultivated on MRS (*Lactobacillus*) or chopped meat broth (*C. bartlettii*; Anaerobe Systems, Morgan Hill, CA, USA) with 25 μg/ml rifampin and incubated overnight at 37°C. Cultures were then subcultured (1%) with 100 μg/ml rifampin and incubated overnight at 37°C. Prior to inoculations, 320 day-of-hatch turkey toms were tagged, weighed, and randomized into 32 cages with each treatment repeated in triplicate. Poults were sampled to confirm absence of rifampin-resistant lactobacilli in their gut microbiota. The next day, poults were inoculated with 0.5 ml of inoculum containing 1 × 10^8^ CFU of each rifampin-resistant strain. At days 3, 7, and 14 postinoculation, 3 birds per cage (9 birds per treatment) were euthanized and their ileum contents were collected. Samples were serially diluted and plated onto MRS or tryptic soy agar plus 5% sheep’s blood containing 100 μg/ml rifampin. Colonies were counted following incubation.

### Probiotic blends.

FM-B11 (Novozymes, Bagsvaerd, Denmark) was used as a commercial poultry-derived probiotic treatment. Strains of L. johnsonii and *L. aviarius* for the T-Pbx group were chosen based on their positive correlation with poultry performance and relative abundance in the context of the developing turkey microbiota (Fig. 1). The final probiotic blend for T-Pbx was created using equal parts of L. johnsonii UMNLJ21 (NCBI accession numbers CP021701, CP021702, and CP021703), *L. aviarius* strains UMNLAv12 and UMNLAv13 (NZ_LWUE00000000 and NZ_LWUF00000000, respectively), and ATCC *C. bartlettii* strain BAA-827 (FUXV00000000).

### Caged experiments comparing antibiotics and probiotics.

Male Hybrid Converter commercial turkey poults were purchased at day of hatch and placed into battery cages. Prior to placement, all birds were weighed, sorted, and placed to normalize average weights at the start of the experiment. Eight caged-bird replicates were included for each of 5 treatment groups, with 10 birds per cage (*n* = 80 birds per treatment, 400 birds total). Treatment groups included a negative control (Control), GroGel carrier control administered daily (Prebx; Dawe’s Nutrition, Arlington Heights, IL, USA), continuous subtherapeutic BMD administration (50 g/ton premixed in feed), commercial probiotic (FM-B11) administered with GroGel carrier, and experimental 4-strain turkey-tailored probiotic (T-Pbx) administered with GroGel carrier (Table 1). GroGel carrier (with or without probiotics) was prepared according to the manufacturer’s instructions. At day of hatch, oral inoculations were delivered to the crop of each turkey with an exact delivery of 1 × 10^8^ CFU of each probiotic mixture. Birds in the Control group were mock inoculated using an empty inoculation tool, and birds in the Prebx and BMD groups were inoculated with 0.5 ml of GroGel carrier. On subsequent days, GroGel with and without probiotic blends was delivered on trays in cages. The volume of GroGel delivered was calculated so that approximately 1 × 10^8^ CFU of probiotic mixture was delivered, on average, to each bird in the cage. Body weights and feed consumption were measured throughout the experiment.

Cecum and ileum collections were performed at day 0 from 30 birds and on days 3, 6, and 13 from 2 birds per cage (16 birds per treatment, 80 birds total). Samples were collected by aseptically squeezing ileum sections and cecal pouches into collection tubes. Samples were stored at −80°C until processing. DNA was extracted from 250 mg of each sample using the Mo Bio PowerSoil-htp 96-well DNA isolation kit (Mo Bio, Carlsbad, CA), according to the manufacturer’s protocol. Total RNA was isolated from each sample by TRIzol extraction (Ambion, Inc., Austin, TX, USA) followed by treatment with DNase (Turbo DNA-free kit; Ambion, Inc.). Each sample was quantified by RiboGreen assay (Invitrogen Corp., Carlsbad, CA, USA), and RNA integrity was confirmed on the 2100 Bioanalyzer (Agilent Technologies, Santa Clara, CA, USA).

Morphometric analysis of ileum samples was performed by excising the distal end of the ileum and placing it immediately into formalin. Twelve birds were sampled per treatment group. From each sample, 4 to 6 transversely cut pieces were embedded and sectioned for hematoxylin and eosin staining. Sections were imaged with a Nikon Eclipse E800 microscope using a 4× lens objective. At least 20 villus heights and crypt depths per sample were measured using the FIJI/ImageJ program, and data were averaged and converted to height/depth ratios.

### Bacterial and fungal community profiling.

DNA isolated from the ileum and cecum was used to amplify the V4 region of the 16S rRNA gene at the University of Minnesota Genomics Center (Minneapolis, MN, USA) using a previously described dual index protocol (36). Ileum DNA was also used to amplify ITS2 using a previously described protocol (37). Amplicons were normalized and pooled for Illumina library construction using the TruSeq Nano kit (Illumina). Sequencing was performed by the University of Minnesota Genomics Center on an Illumina MiSeq using 2- by 250-bp paired-end V2 or 2- by 300-bp V3 MiSeq reagent kits (Illumina).

16S rRNA amplicon sequences were processed with SHI7 using the Nextera adaptor trimming option (38). NINJA-OPS was used to align the preprocessed reads against the Greengenes 97 database (39) using the more sensitive max mode (39). The resulting OTU table was filtered to keep only samples with at least 1,500 sequences and OTUs in at least 5% of the samples. A collapsed taxon table was converted to relative abundance, and taxa representing less than 10% of the sample were designated Other. Beta diversity metrics assessed were Bray-Curtis and UniFrac. Distance matrices were calculated for each metric using the rarefied version of the filtered OTU table (10,000 sequences) with phyloseq (unweighted UniFrac) (40) and vegan packages in R (Bray-Curtis) and in QIIME v1.8.0 (unweighted UniFrac, weighted UniFrac, and Bray-Curtis). Alpha diversity metrics were calculated using the rarefied OTU table in QIIME v1.8.0 (41). To test for differentially abundant OTUs and between-omics (bacterial-fungal, bacterium-transcriptome) correlations, a centered log-ratio (CLR)-transformed version of the filtered OTU table was created in R.

ITS2 sequences were processed with SHI7 using the TruSeq3-2 adaptor trimming option (38). NINJA-OPS was used to align preprocessed reads against the UNITE v7 singleton-exclusive dynamic (31 January 2016) fungal ITS database release for NINJA-OPS using default options (39). The resulting OTU table was filtered to keep only samples with at least 50 aligned reads and OTUs occurring in at least 5% of the samples. Taxon summaries were created in R, collapsed taxon tables were converted to relative abundance, and taxa representing less than 20% of the sample were designated Other. Bray-Curtis distances and alpha diversities were calculated after converting the filtered OTU table to relative abundance in QIIME v1.8.0 (41). To test for differentially abundant OTUs and fungal-bacterial correlations, a CLR-transformed version of the filtered OTU table was created in R.

### Statistical analyses.

Differences in turkey weights and morphometric measurements were analyzed using a one-way analysis of variance (ANOVA) with Tukey honestly significant difference (HSD) for *post hoc* testing. For beta diversity metrics, principal-coordinate analyses were performed in R using the vegan package (42), and differences in centroids were tested with permutational multivariate analysis of variance (PERMANOVA) (Adonis) from the vegan package, either using all groups or pairwise. Student’s *t* tests were used for calculating differentially abundant OTUs from CLR-transformed OTU relative abundances, as well as for calculating differences in alpha diversity metrics, weight, and normalized reactome pathway expression. Procrustes analyses were performed in R, and significance was tested with 999 permutations and a Mantel test from the vegan package (42). Between-omic correlations were calculated using Pearson’s correlations with false-discovery rate correction of CLR-transformed abundances (bacteria and fungi) and normalized transcriptome data (microbiome package).

### RNA-Seq of turkey host transcriptome.

Replicate ileum samples were sequenced from each treatment group (*n* = 4). Indexed libraries were constructed with 1 μg of total RNA/sample with the TruSeq RNA sample preparation kit version 2 (Illumina) and size selected for approximately 200-bp inserts. Libraries were multiplexed, pooled, and sequenced over 2 lanes on the HiSeq 2500 using v3 chemistry (Illumina) to produce 125-bp paired-end reads. Sequence adaptors were removed, and low-quality bases were trimmed using Trimmomatic (32). Quality control checks on raw sequence data for each sample were performed with FastQC (43). Read mapping was performed via Bowtie (v2.2.4.0) using the turkey genome (UMD 5.0, NCBI annotation 102). Read counts were normalized by dividing total read counts by the group sample sum. Euclidean hierarchical clustering of samples was performed using normalized read counts. Empirical analysis of differential gene expression and ANOVA was performed on EdgeR-normalized read counts (Bonferroni and FDR corrected). Pairwise comparisons between treatment groups were made in the Bioconductor (3.2) R package DESeq2 (44). Genes with significant differential expression were used to investigate affected gene pathways using Ingenuity Pathway Analysis (IPA) (Ingenuity Systems, Redwood City, CA, USA). Gene enrichment tests were performed using the PANTHER Overrepresentation Test (GO Consortium release 20150430, http://geneontology.org/). For reactome pathway analysis, normalized transcript expression levels associated with each pathway were summed for each sample using the Reactome Pathways Gene Set (accessed June 2017) (45). Euclidean distances of the reactome pathways for each sample were calculated using the vegan package in R.

### Kinome arrays.

For kinome arrays, 40-mg tissue samples were flash frozen in liquid nitrogen and subsequently stored at −80°C until processing. Samples were thawed and homogenized using 1.4-mm ceramic beads (Omni International, Kennesaw, GA, USA) with lysis buffer (100 μl; Sigma-Aldrich, St. Louis, MO, USA) in a Bead Ruptor 24 (Omni International) for two 10-s cycles at machine speed 6. Homogenized tissue was spun in a microcentrifuge at 21,200 × *g* for 10 min at 4°C, and array protocols were carried out as previously described (46). After incubation for 2 h at 37°C, arrays were washed with shaking sequentially in phosphate-buffered saline (PBS) plus 1% Triton, twice in 2 M NaCl plus 1% Triton, and a final wash in distilled, deionized H_2_O. Arrays were scanned using a Tecan PowerScanner microarray scanner (Tecan Systems, San Jose, CA, USA) at 532 to 560 nm with a 580-nm filter to detect the phosphostain dye fluorescence. Images were gridded using GenePix Pro 7 software (San Jose, CA, USA), and spot intensity signal was collected. The data from GenePix were then analyzed by the PIIKA2 peptide array analysis software (47).

### Pen trials to validate T-Pbx effects in turkeys.

A pen trial with 7 replicates per experimental group was performed. Four experimental groups were included: control, T-Pbx, T-Pbx plus 1% lactose in feed, and T-Pbx plus 0.5 lb/ton SafMannan (Phileo by Lesaffre, Marcq-en-Baroeul, France) in feed as a yeast-based prebiotic. Twenty-four commercial turkey poults per pen were placed at day of hatch following sorting weights to normalize treatment groups. Prior to placement, birds in T-Pbx groups were orally gavaged with 1 × 10^8^ CFU of probiotic mixture, as described above. Birds were reinoculated with T-Pbx again at 1, 2, and 3 weeks of age. Bird weights were obtained at 1, 2, 3, 6, 9, 12, and 15 weeks of age. Birds were supplied with feed and water *ad libitum*. The experiment was terminated at 15 weeks of age.

### Data availability.

Raw reads from 16S rRNA gene amplicon sequencing, ITS2 amplicon sequencing, and host RNA-Seq are deposited under BioProject accession number PRJNA490872 at NCBI. Raw reads from bacterial isolate sequencing are deposited under BioProject accession numbers PRJNA316009 and PRJNA316010.
