# Supplementary figures and images for: Antibiotics and Host-Tailored Probiotics Similarly Modulate Effects on the Developing Avian Microbiome, Mycobiome, and Host Gene Expression
Source: mBio. 2019 Oct 15;10(5):e02171-19. doi: 10.1128/mBio.02171-19 (PMC6794479; doi:10.1128/mBio.02171-19)

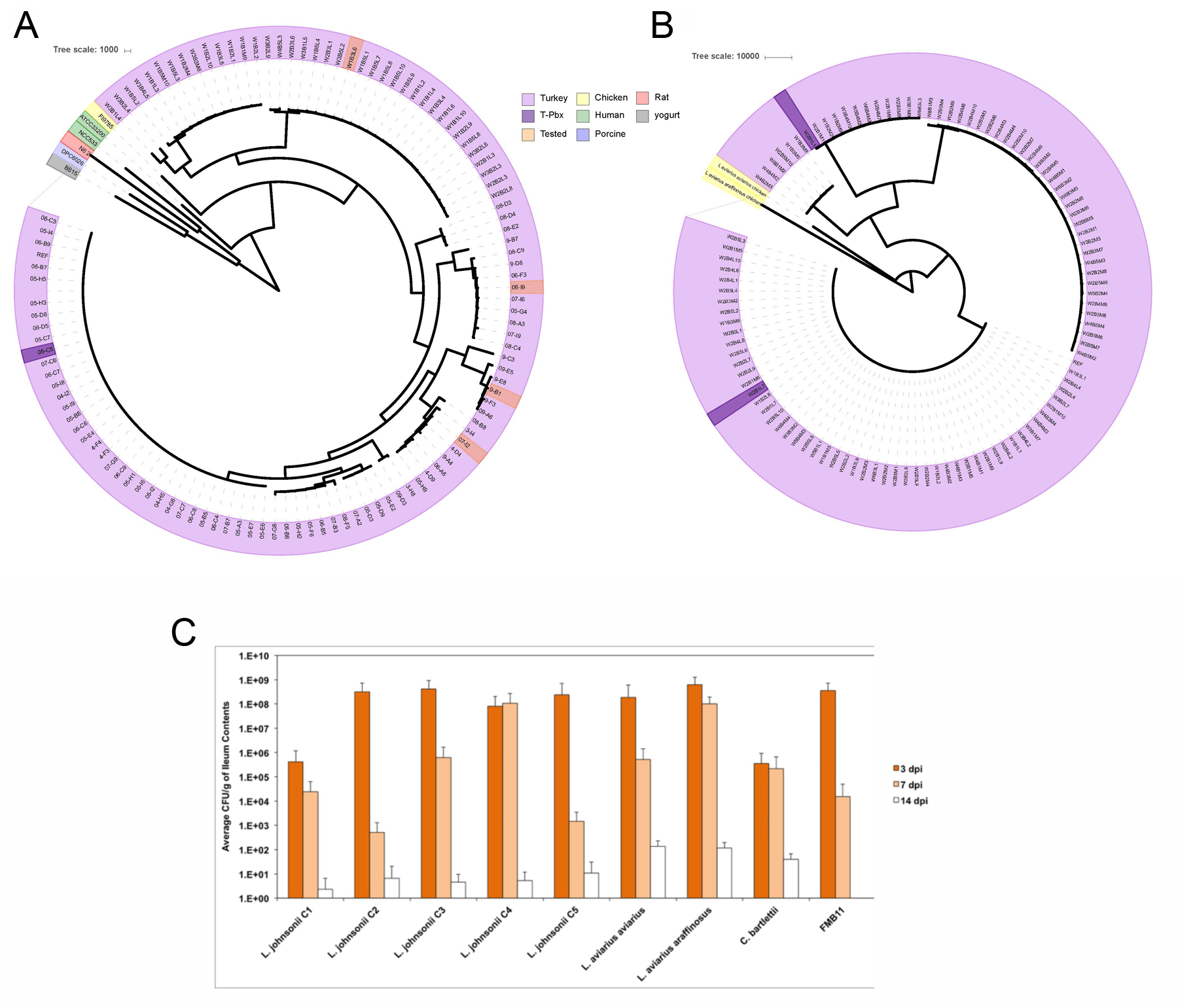

Supplement: FIG S1 [file mBio.02171-19-sf001.jpg]

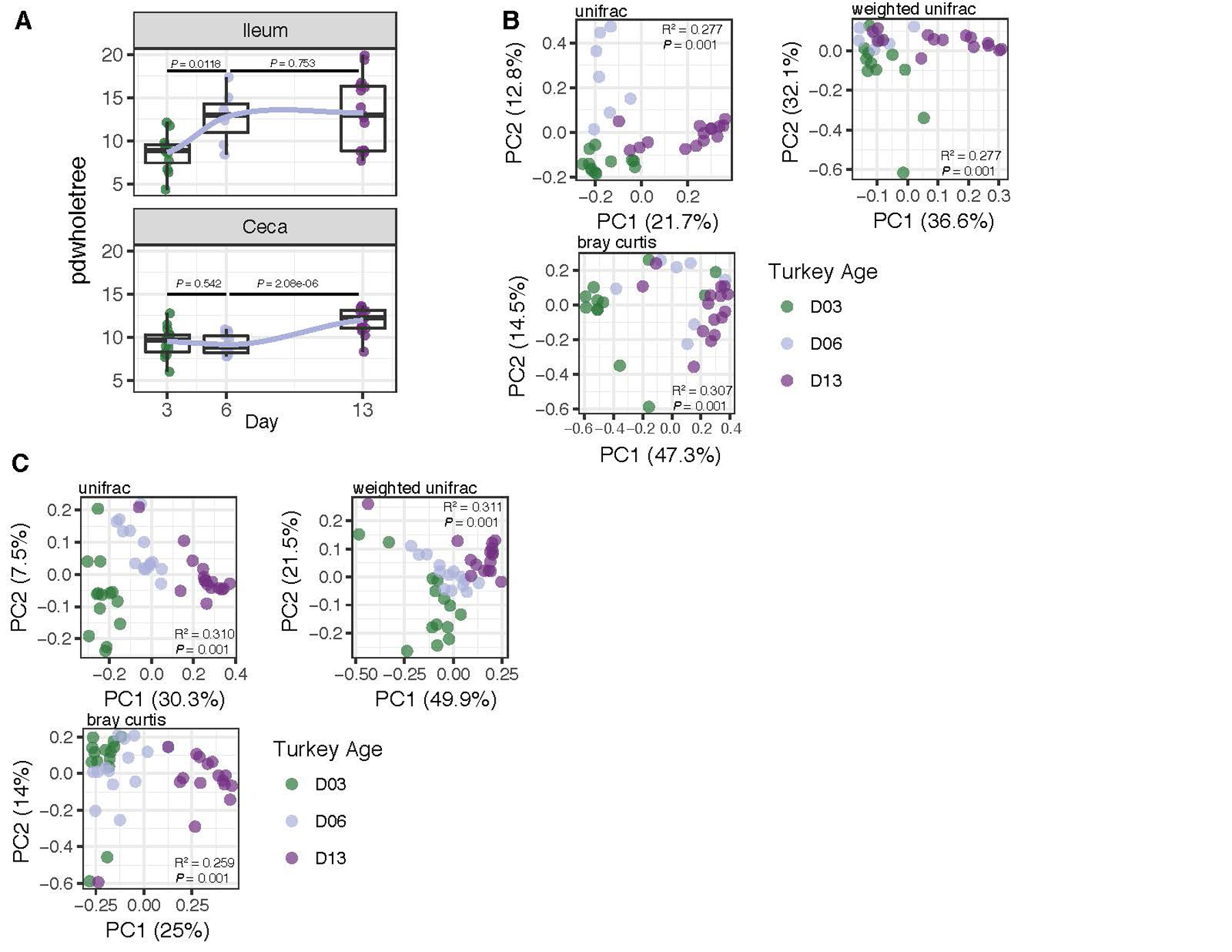

Supplement: FIG S2 [file mBio.02171-19-sf002.jpg]

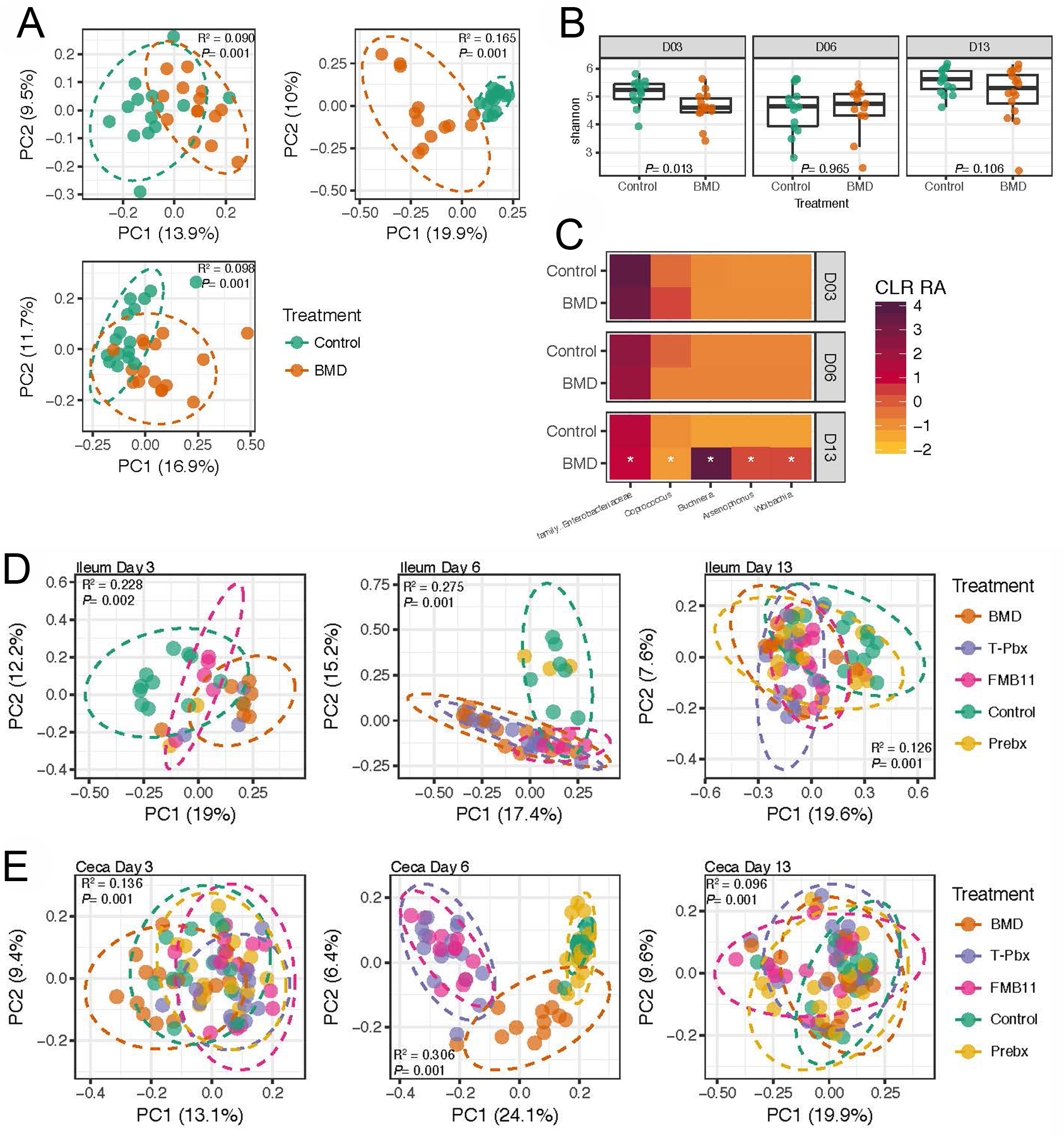

Supplement: FIG S3 [file mBio.02171-19-sf003.jpg]

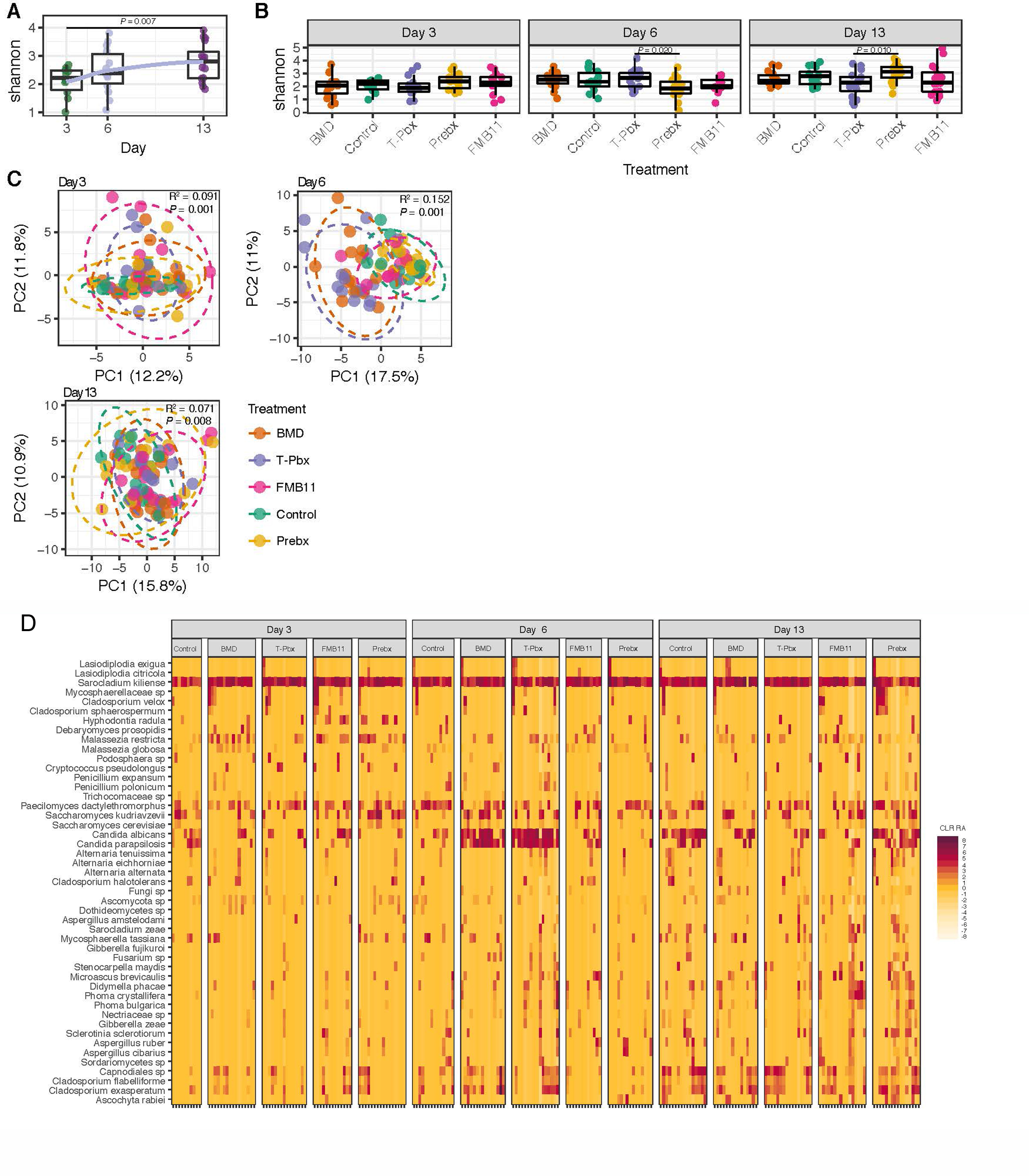

Supplement: FIG S4 [file mBio.02171-19-sf004.jpg]

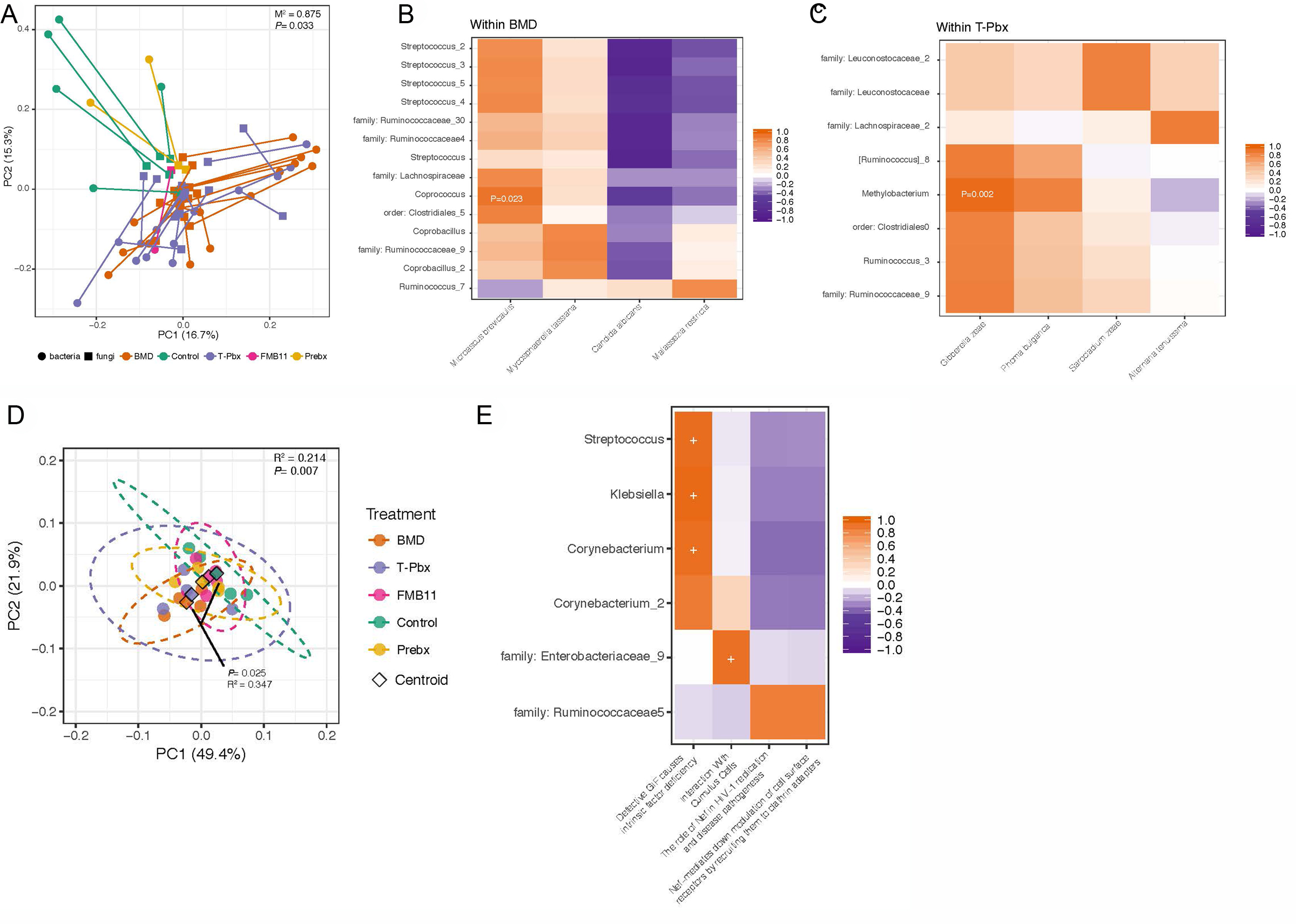

Supplement: FIG S5 [file mBio.02171-19-sf005.jpg]
